# Cariprazine’s efficacy in treating depressive symptoms – pooled data from schizophrenia, bipolar depression and major depression trials

**DOI:** 10.1192/j.eurpsy.2023.664

**Published:** 2023-07-19

**Authors:** R. S. McIntyre, R. Csehi, G. Németh

**Affiliations:** ^1^Psychiatry and Pharmacology, University of Toronto, Toronto, Canada; ^2^Gedeon Richter Plc., Budapest, Hungary

## Abstract

**Introduction:**

Depressive symptoms are a common feature of schizophrenia (SCH) and define bipolar disorder and major depressive disorder (MDD). Their emergence is related to altered neurotransmission at the serotonin receptors and potentially at dopamine D3 receptors.

**Objectives:**

The aim of this analysis was to examine the efficacy of cariprazine (CAR) in treating depressive symptoms in SCH, bipolar depression (BD) and MDD.

**Methods:**

Clinical trials with randomised, double-blind, placebo (PLB)-controlled designs were included in these analyses. Data from 3 SCH [NCT00694707, NCT01104766, NCT01104779; 1.5-9 mg/d] and 3 BD [NCT01396447, NCT02670538, NCT02670551; 1.5-3 mg/d] studies were pooled. In MDD, add-on CAR to antidepressant treatment was evaluated against PLB in two studies [NCT03738215: 1.5 and 3 mg/d; NCT01469377: 1-2 mg/d and 2-4.5 mg/d).

Least square (LS) mean changes were analysed using Mixed Model Repeated Measures: from baseline (BL) to Week 6 in the Positive and Negative Syndrome Scale (PANSS)-derived Marder anxiety/depression factor items (schizophrenia); from BL to Week 6 in the Montgomery-Åsberg Depression Rating Scale (MADRS) total scores (bipolar depression); and from BL to Week 6 [NCT03738215] and Week 8 [NCT01469377] in MADRS total score (major depressive disorder).

**Results:**

Altogether, 1466 SCH (PLB=442, CAR=1024) patients were included in the pooled analysis. In the BD analysis, data from 1383 (PLB=460, CAR=923) patients were pooled. In the MDD trials, there were 502 CAR (1.5mg/d=250, 3 mg/d=252) and 249 PLB-treated patients [NCT03738215], and 544 CAR (1-2 mg/d=273, 2-4.5 mg/d=271) and 264 PLB patients in the other study [NCT01469377]. In SCH, CAR achieved significantly greater reductions than PLB on the Marder anxiety/depression factor domain (LS mean change: PLB= -2.66, CAR= -3.26, p<0.01): the effect was driven by 3 out of 4 items. In BD, CAR yielded significantly greater improvement on the MADRS compared to PLB (LS mean change: PLB= -12.05, CAR= -14.69, p<0.001), which was driven by 9 out of 10 items. In MDD [NCT03738215], CAR 1.5 mg/d add-on significantly alleviated depressive symptoms compared to PLB (LS mean change: PLB= -11.5, CAR 1.5mg/d= -14.1, p<0.01), while in the other MDD trial [NCT01469377], CAR 2-4.5 mg/d add-on produced significantly greater reductions than PLB (LS mean change: PLB= -12.5, CAR 2-4.5 mg/d= -14.6, p<0.01).

**Conclusions:**

These findings indicate that CAR is an effective treatment option for the treatment of depressive symptoms independent of disease (in SCH, BD and MDD), being a transdiagnostic broad-spectrum treatment option.

**Disclosure of Interest:**

R. McIntyre Grant / Research support from: CIHR/GACD/National Natural Science Foundation of China (NSFC), the Milken Institute, Consultant of: Lundbeck, Janssen, Alkermes, Neumora Therapeutics, Boehringer Ingelheim, Sage, Biogen, Mitsubishi Tanabe, Purdue, Pfizer, Otsuka, Takeda, Neurocrine, Sunovion, Bausch Health, Axsome, Novo Nordisk, Kris, Sanofi, Eisai, Intra-Cellular, NewBridge Pharmaceuticals, Viatris, Abbvie, Gedeon Richter, Recordati, Atai Life Sciences, Speakers bureau of: Lundbeck, Janssen, Alkermes, Neumora Therapeutics, Boehringer Ingelheim, Sage, Biogen, Mitsubishi Tanabe, Purdue, Pfizer, Otsuka, Takeda, Neurocrine, Sunovion, Bausch Health, Axsome, Novo Nordisk, Kris, Sanofi, Eisai, Intra-Cellular, NewBridge Pharmaceuticals, Viatris, Abbvie, Gedeon Richter, Recordati, Atai Life Sciences, R. Csehi Employee of: Gedeon Richter Plc., G. Németh Employee of: Gedeon Richter Plc.

